# Synchronized Molecular-Dynamics Simulation of the Thermal Lubrication of an Entangled Polymeric Liquid

**DOI:** 10.3390/polym11010131

**Published:** 2019-01-13

**Authors:** Shugo Yasuda

**Affiliations:** Graduate School of Simulation Studies, University of Hyogo, Kobe 650-0047, Japan; yasuda@sim.u-hyogo.ac.jp

**Keywords:** multiscale modeling, polymeric liquid, entanglement, viscous heating, lubrication

## Abstract

The thermal lubrication of an entangled polymeric liquid in wall-driven shear flows between parallel plates is investigated by using a multiscale hybrid method, coupling molecular dynamics and hydrodynamics (i.e., the synchronized molecular dynamics method). The temperature of the polymeric liquid rapidly increases due to viscous heating once the drive force exceeds a certain threshold value, and the rheological properties drastically change at around the critical drive force. In the weak viscous-heating regime, the conformation of polymer chains is dominated by the flow field so that the polymers are more elongated as the drive force increases. However, in the large viscous-heating regime, the conformation dynamics is dominated by the thermal agitation of polymer chains so that the conformation of polymers recovers more uniform and random structures as the drive force increases, even though the local shear flows are further enhanced. Remarkably, this counter-intuitive transitional behavior gives an interesting re-entrant transition in the stress–optical relation, where the linear stress–optical relation approximately holds even though each of the macroscopic quantities behaves nonlinearly. Furthermore, the shear thickening behavior is also observed in the large viscous-heating regime—this was not observed in a series of previous studies on an unentangled polymer fluid. This qualitative difference of the thermo-rheological property between the entangled and unentangled polymer fluids gives completely different velocity profiles in the thermal lubrication system.

## 1. Introduction

Polymeric fluids exhibit complicated flow behaviors because the microscopic dynamics of polymer chains are highly correlated with global hydrodynamic transport [1]. In particular, in high-speed devices, the microscopic dynamics are significantly affected by viscous heating because the polymeric fluid has a large Prandtl number, so the microscopic dynamics and the hydrodynamic heat and momentum transports are mutually correlated. Predicting the microscopic dynamics and the macroscopic thermal flows in high-speed devices is challenging from both scientific and engineering points of view. Computer simulations are expected to be useful to solve these mutually correlated multiscale systems.

Multiscale simulations to tackle the flow behaviors of complex fluids have been developed by various researchers. The pioneering research was carried out by Laso and Öttinger [2,3,4]. They proposed the CONNFFESSIT approach for polymeric liquids, where the local stress in the fluid solver is calculated by a microscopic simulation without using any constitutive relations. The strategy exploited in the CONNFFESSIT approach was also introduced into heterogeneous multiscale modeling (HMM), which was first put forward by E and Enquist [5]. The HMM method has been applied to various problems [6,7,8]. The equation-free method proposed by Kevrekids et al. is also based on a similar idea and has been applied to various problems [9,10].

The scale-bridging method to reproduce the memory effect of polymer dynamics was first developed by De et al. [11]. They succeeded in reproducing the nonlinear viscoelastic behavior of a polymeric liquid in terms of the slab and cylindrical geometries [11,12]. The multiscale simulation of polymeric flows with the advection of memory in two and three dimensions was developed by Murashima and Taniguchi [13,14,15]. Noise reduction algorithms, filtering the microscopic particle-based simulation data in hybrid multiscale modeling, were also investigated in Ref. [16].

We have also developed multiscale simulations which couple the molecular dynamics and hydrodynamic transports. The method was first developed for a simple Lennard-Jones fluid [17] and subsequently extended to polymeric liquids with the memory effect [18,19,20,21]. Recently, we proposed the synchronized molecular dynamics (SMD) method for thermal lubrication flows of polymeric liquids, in which the local viscous heating is autonomously generated in the local MD cell according to the local shear flow. The local MD cells are also synchronized to satisfy the global heat and momentum transports [22].

The SMD method assumes that the molecular states are homogeneous in local fluid elements and the momentum and energy transports between the fluid elements are described by the hydrodynamic equations. Although this assumption discards some part of the accuracy of a full molecular description, it gives a great advantage in computational efficiency when one considers, for example, the thermal flows of locally homogeneous polymer fluids at a macroscopic scale far beyond the molecular size.

In this paper, we tackle the thermal lubrication of entangled polymer chains by using the SMD method. This study is the extension of a series of previous studies on an unentangled polymer fluid [22,23,24] to the entangled polymer fluid. In the previous studies, a counter-intuitive transitional behavior induced by viscous heating was discovered in the dynamics of polymer conformation. The robustness of the linear stress-optical relation was also confirmed even in the complicated transitional behavior. It is well known that the entangled polymer fluid has distinctive features in the relaxation dynamics and rheological property [25,26,27,28]. In this study, the common properties observed both in entangled and unentangled polymer fluids and some particular properties of the entangled polymer fluid are clarified.

In the following, we first describe the problem and the model polymeric liquid considered in this paper. Here, we also clarify the intrinsic properties in the relaxation dynamics of the entangled polymer chains in comparison to those described by the reptation theory [25]. The SMD method is described in Section 3 and the results are presented and discussed in Section 4. Finally we give a brief summary.

## 2. Problem

### 2.1. Geometry

**Figure 1:**
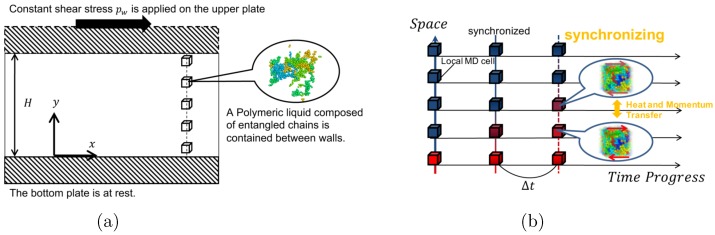
Schematics of (**a**) the geometry of the problem and (**b**) the time schedule scheme of synchronized molecular dynamics simulation.

We consider a polymeric liquid confined between parallel walls, as in Figure 1. The upper wall starts to move in the *x*–direction at time t=0 by an applied shear stress $p_{w}$, while the temperature of the walls $T_{w}$ is kept constant.

The polymeric liquid is initially in a quiescent state with uniform density $\rho_{0}$ and temperature $T_{0}$, where the initial temperature of the polymeric liquid is the same as the wall temperature (i.e., $T_{0}=T_{w}$).

The polymeric liquid starts to deform the uniform quiescent state at t=0 and forms non-uniform distributions of velocity and temperature via the heat and momentum transfers described by

(1a) $$\begin{array}{cc} \rho_{0}\frac{\partial v_{x}}{\partial t} & =\frac{\partial p_{xy}}{\partial y}, \end{array}$$

(1b) $$\begin{array}{cc} \rho_{0}\frac{\partial e}{\partial t} & =p_{xy}\dot{\gamma }+\lambda \frac{\partial^{2}T}{\partial y^{2}}, \end{array}$$

where $v_{\alpha }$ is the velocity, $p_{\alpha \beta }$ is the stress tensor, *e* is the internal energy per unit mass, and $\dot{\gamma }$ is the shear rate (i.e., $\dot{\gamma }=\partial v_{x}/\partial y$). Hereafter, the subscripts α, β, and γ represent the index of Cartesian coordinates (i.e., {α,β,γ}∈{x,y,z}). Here, we assume the one-dimensional incompressible flow, where the macroscopic quantities are uniform in the *x*– and *z*–directions, ∂/∂x = ∂/∂z = 0, and the density of the polymeric liquid is constant. Fourier’s law for heat flux with a constant and uniform thermal conductivity λ is also considered in Equation (1b).

We also consider the non-slip and non-jump boundary conditions of velocity and temperature for Equation (1). That is, the velocity and temperature of the polymeric liquid on the wall are the same as those of the wall.

### 2.2. Model Polymeric Liquid

The polymeric liquid is composed of so-called Kremer–Grest chains of 250 beads, where each bead particle interacts via the repulsive part of the Lennard-Jones potential,

(2) $$U_{\mathrm{LJ}}\left(r\right)=\left\{\begin{array}{cc} 4\epsilon \left[{\left(\frac{\sigma }{r}\right)}^{12}-{\left(\frac{\sigma }{r}\right)}^{6}\right]+\epsilon , & \;(r\leq 2^{1/6}\sigma ),\\ 0, & \;(r\geq 2^{1/6}\sigma ), \end{array}\right.$$

and consecutive beads on each chain are connected by an anharmonic spring potential,

(3) $$U_{F}\left(r\right)=-\frac{1}{2}k_{c}R_{0}^{2}\ln\left[1-{\left(\frac{r}{R_{0}}\right)}^{2}\right],$$

where $k_{c}$ = 30$\epsilon /\sigma^{2}$ and $R_{0}$ = 1.5σ [26]. Hereafter, unless otherwise stated, we measure the physical quantities with the units of length σ, time $\sqrt{m\sigma^{2}/\epsilon }$, and temperature $\epsilon /k_{B}$, where *m* is the mass of the bead particle and $k_{B}$ is Boltzmann’s constant.

In this study, we only consider a polymeric liquid with the initial density $\rho_{0}=0.85$ and initial temperature $T_{0}=0.4$. Please note that the wall temperature $T_{w}=0.4$ and the number of beads in each polymer chain $N_{b}=250$ are fixed. With this bead number $N_{b}=250$, the model polymeric liquid shows the characteristic behaviors of entangled polymer chains in the relaxation dynamics.

**Figure 2:**
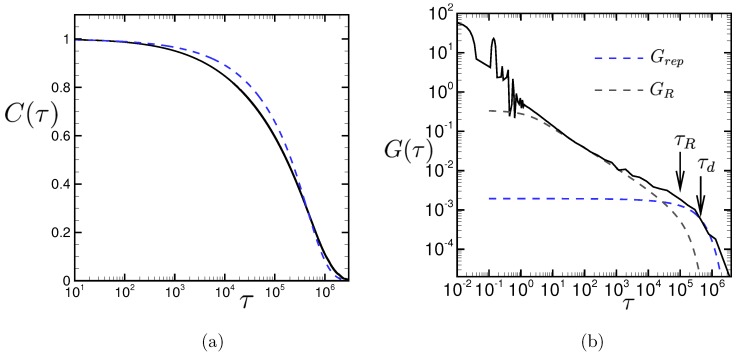
(**a**) The time-correlation function of the end-to-end vector of polymer chain C(τ) defined in Equation (4) and (**b**) the stress relaxation function G(τ) defined in Equation (5). In (**a**), the blue dashed line shows the theoretical formalism of the reptation dynamics with the relaxation time $\tau_{d}=4.4\times 10^{5}$. In (**b**), the theoretical formalism of the stress relaxation function for the Rouse dynamics, $G_{R}\left(\tau \right)$ in Equation (7), and for the reptation dynamics, $G_{rep}\left(\tau \right)$ in Equation (8), are also plotted.

Figure 2 shows the normalized time-correlation function of the end-to-end vector of polymer chain C(τ), which is calculated as

(4) $$C\left(\tau \right)=\frac{\langle \bar{P\left(\tau +t_{0}\right)\cdot P\left(t_{0}\right)}\rangle }{\langle \bar{|P\left(t_{0}\right){|}^{2}}\rangle },$$

and the stress relaxation function G(τ), which is calculated as

(5) $$G\left(\tau \right)=\frac{T_{0}}{V}\bar{p_{xy}\left(\tau +t_{0}\right)p_{xy}\left(t_{0}\right)}$$

for the present model polymeric liquid. Here, P(t) represents end-to-end vectors of each polymer chain, $p_{xy}\left(t\right)$ is the macroscopic shear stress, which is the average of the microscopic shear stresses of each bead particle in the simulation box, and *V* is the volume of the simulation box. We also write $\bar{X(t_{0})}$ as the average of *X* with respect to $t_{0}$ and $\langle X\rangle$ as the ensemble average of *X* over all chains.

In Figure 2a, our numerical result of C(τ) is compared with a theoretical formalism, which is obtained in both the Rouse dynamics and the reptation dynamics,

(6) $$C_{r}\left(\tau \right)=\sum_{\mathrm{odd}p}\frac{8}{\pi^{2}p^{2}}\exp\left(-p^{2}\tau /\tau_{d}\right),$$

where the summation is over odd *p* and $1\leq p\leq N_{b}-1$ [25].

The relaxation time $\tau_{d}=4.4\times 10^{5}$ is measured by fitting Equation (6) with our numerical result at $\tau =\tau_{d}$ (i.e., $C\left(\tau =\tau_{d}\right)=C_{r}\left(\tau_{d}\right)$). We note that the relaxation time $\tau_{d}$ corresponds to the disengagement time $\tau_{d}$ of entangled polymer chains if we assume that the time correlation function is described by the reptation dynamics.

In Figure 2b, the numerical result of the stress relaxation function G(τ) is compared with the theoretical formalism for the Rouse dynamics $G_{R}\left(\tau \right)$ and for the reptation dynamics $G_{rep}\left(\tau \right)$ which are written, respectively, as

(7) $$G_{R}\left(\tau \right)=\frac{\rho_{0}T_{0}}{N_{b}}\sum_{p=1}^{N_{b}-1}\exp\left(-p^{2}t/\tau_{R}N_{b}^{2}\right),$$

and

(8) $$G_{rep}\left(\tau \right)=\frac{\rho_{0}T_{0}}{N_{e}}C_{r}\left(\tau \right).$$

Here, the Rouse relaxation time $\tau_{R}=1.0\times 10^{5}$ and the average number of beads between entanglements $N_{e}=175$ are estimated from our numerical results. Numerical results of $\tau_{d}$ and Ne for the Kremer–Grest chains with different parameters can be found in Ref. [28] and the references therein.

The average number of beads between entanglements is measured by fitting the formula Equation (8) with our numerical result at $\tau =\tau_{d}$. The Rouse relaxation time $\tau_{R}$ is calculated by the formula $\tau_{d}/\tau_{R}=3N_{b}/N_{e}$. In Figure 2, it can be seen that the relaxation dynamics of the model polymeric liquid is well-described by the reptation dynamics for entangled polymer chains at long times (i.e., $\tau >\tau_{r}$). The transition of the Rouse dynamics to the reptation dynamics can also be clearly observed in the stress relaxation function. Incidentally, the oscillation of G(τ) in τ≲1 in Figure 2b is due to the spring potential between the consecutive bead particles in the same polymer chain.

The viscosity of the model polymeric liquid in the quiescent state $\eta_{0}=980$ is calculated from the stress relaxation function G(τ) as $\eta_{0}=\int_{0}^{\tau_{\infty }}G\left(\tau \right)d\tau$. The integral with respect to τ is almost saturated at $\tau_{\infty }=2\times 10^{6}$ and the deviation of $\eta_{0}$ in the period for $\tau_{\infty }=2\times 10^{6}$ to 5 $\times 10^{6}$ is at most 8%.

## 3. Simulation Method

We investigate the thermal lubrication of the polymeric liquid composed of entangled chains by using the synchronized molecular dynamics (SMD) simulation [22]. In the SMD method, the gap between the upper and lower walls is uniformly divided into *M* mesh intervals with Δy=H/M, and small sub cells are assigned to each mesh interval, where the molecular dynamics of local fluid elements are calculated. (See Figure 1) In each sub-MD cell, the nonequilibrium molecular dynamics (NEMD) simulations are performed independently in the time interval Δt, using the SLLOD algorithm [29,30] according to the local shear rate $\dot{\gamma }=\frac{\partial v_{x}}{\partial y}$, which is calculated by the global momentum transport Equation (1a). In the NEMD simulations, the viscous heating due to the local shear flow (i.e., the first term of the right-hand side of Equation (1b)) is autonomously calculated without using any thermostat algorithms. Thus, the local temperatures increase in each sub-MD cell in the time interval Δt.

Because each NEMD simulation is performed independently, the sub-MD cells have to be corrected to satisfy the global heat and momentum transports including the boundary conditions at certain times. In the SMD method, at every time interval Δt, the local sub-MD cells are synchronized via the global heat and momentum transport Equations (1).

Using the time average of the instantaneous shear stress $P_{xy}$ calculated in each sub-MD cell, the local shear stress at time t=nΔt, ${\dot{\gamma }}^{n}\left(y\right)$ is calculated by the following equation,

(9) $$v_{x}^{n}\left(y\right)=v_{x}^{n-1}\left(y\right)+\frac{1}{\rho_{0}}\frac{\partial }{\partial y}\int_{(n-1)\Delta t}^{n\Delta t}P_{xy}\left(s;{\dot{\gamma }}^{n-1}\left(y\right)\right)ds,$$

and ${\dot{\gamma }}^{n}\left(y\right)=dv_{x}^{n}\left(y\right)/dy$. Here, the superscript *n* represents the time step number, $P_{xy}\left(s;{\dot{\gamma }}^{n-1}\left(y\right)\right)$ is the instantaneous shear stress in the NEMD simulation with the shear rate ${\dot{\gamma }}^{n-1}\left(y\right)$, and *s* is the time in the NEMD simulation.

**Figure 3:**
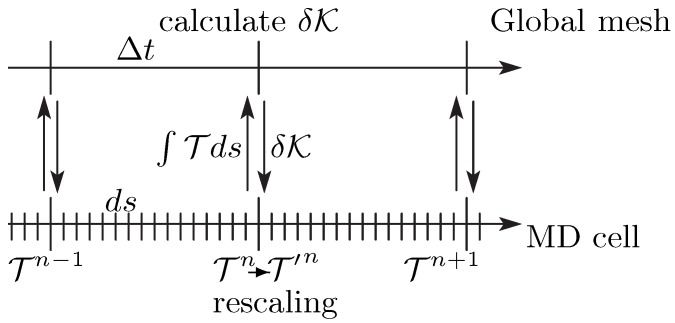
The time schedule scheme for the calculation of the temperature in the SMD method. The upper side represents the progress on the global mesh system, and the lower side represents the progress in the sub-MD cell. The time interval of the synchronization Δt=1 and the time-step size of the MD simulation ds=0.0025 are fixed.

The kinetic energies of each sub-MD cell are also corrected according to the heat fluxes between neighbor MD cells to satisfy the global energy transport Equation (1b). That is, by using the heat flux calculated by

(10) $$\delta K=\frac{\lambda }{\rho_{0}}\frac{\partial^{2}}{\partial y^{2}}\int_{(n-1)\Delta t}^{n\Delta t}T\left(s\right)ds,$$

where T is the instantaneous temperature of the sub-MD cell, the molecular velocities in each sub-MD cell are rescaled according to the corrected temperature $T^{\prime }$, i.e.,

(11) $$T^{\prime }=T+\frac{2}{3}\delta K,$$

at every time interval Δt. (See also Figure 3).

Then, the local MD simulations are resumed by using the rescaled molecular velocities and the new local shear rate ${\dot{\gamma }}^{n}\left(y\right)$ for s=[nΔt,(n+1)Δt]. Please note that the molecular conformation obtained in the previous time interval is succeeded in the new time interval. Thus, the temporal evolution of the microscopic configuration is traced at the microscopic level with time-step size ds to reproduce the memory effect due to the slow dynamics of polymer chains.

Without using the thermostat algorithm, the increase in the local temperature due to the viscous heating, which is significant for polymer fluids, is autonomously calculated in the MD simulation. This is an important advantage of the SMD method because the equation of the time evolution of temperature is unknown for polymer fluids in general since the second term of the right-hand side of Equation (1b), $p_{xy}\dot{\gamma }$ contributes to both temperature and free energy related to the polymer conformation. In the SMD method, the increase in temperature and the change of the conformation of polymers are autonomously generated in the molecular dynamics simulations without using any constitutive equations. Furthermore, local heat generation is conducted to the surrounding fluid to satisfy the global energy transport although the Fourier law of the heat conduction is artificially assumed.

The validity of the SMD method was investigated in the previous study [19,22], where the effects of the time interval Δt, the mesh interval Δx, and the number of particles in each MD cell were investigated in detail. It was clarified that (i) the time interval Δt must be smaller than the viscous diffusion time $\Delta x^{2}/2\eta$, where η is the local viscosity and (ii) the temperature profile is strongly affected by the noise arising from the local MD cell; in order to reduce the noise, one has to increase the number of particles in each MD cell $N_{\mathrm{MD}}$ and keep the ratio of the mesh interval Δx to the size of MD cell $l_{\mathrm{MD}}$, $\Delta x/l_{\mathrm{MD}}$ not too large. The SMD method gives a good balance between the accuracy and computational efficiency when the number of particles in each MD cell is sufficiently large, say $N_{\mathrm{MD}}≳10^{3}$ and the ratio of the mesh interval to the size of MD cell is set as, say, $5≲\Delta x/l_{\mathrm{MD}}≲10$.

## 4. Results and Discussion

The SMD simulation is performed for the geometry shown in Figure 1. At time t=0, the shear stress $p_{w}$ is applied on the upper wall and the upper wall starts to move in the *x*–direction. The upper wall drives the polymeric liquid, and spatial profiles of the velocity $v_{x}\left(y\right)$, temperature T(y), and polymer conformation (i.e., the bond-orientation tensor) $Q_{\alpha \beta }\left(y\right)$ are created between the walls.

The local bond-orientation tensors $Q_{\alpha \beta }\left(y\right)$ are calculated in each MD cell as

(12) $$Q_{\alpha \beta }=\frac{1}{N_{p}}\sum_{\mathrm{chains}}\frac{1}{N_{b}-1}\sum_{j=1}^{N_{b}-1}\frac{b_{j\alpha }}{b_{\min}}\frac{b_{j\beta }}{b_{\min}},$$

where $N_{p}$ is the number of polymer chains in each MD cell, $b_{j}$ for $1\leq j\leq N_{b}-1$ is the bond vector between consecutive beads in the same chain, and $b_{\min}$ is the distance at which the sum $U_{\mathrm{LJ}}\left(r\right)+U_{F}\left(r\right)$ has a minimum (i.e., $b_{\min}≃0.97$).

The initial density and temperature, $\rho_{0}=0.85$ and $T_{0}=0.4$, the wall temperature $T_{w}=0.4$, and the channel width H=5000 are fixed, while the drive force on the upper wall is varied as $p_{w}=$ 0.0005, 0.001. 0.002. 0.005, 0.01, 0.02, 0.03, 0.05, 0.07, and 0.09. Hereafter, the notations $p_{w}$ and $p_{xy}$ denote the drive force and shear stress divided by the density $\rho_{0}$, respectively.

In the numerical scheme, the number of mesh intervals M=32, that is, Δy=156.25, the time interval in Equation (9) Δt=1, the time-step size of MD simulation $\Delta t_{\mathrm{MD}}=0.0025$, and the number of polymer chains in each MD cell $N_{p}=32$ are also fixed. Thus, $N_{b}\times N_{p}=8000$ bead particles are included in each MD cell with the side length $l_{\mathrm{MD}}=21$ and the ratio of the mesh interval Δy to the side length $l_{\mathrm{MD}}$ is $\Delta y/l_{\mathrm{MD}}=7.4$. We also fix the thermal conductivity in Equation (10) as $\lambda /\rho_{0}=100$.

### 4.1. Spatial Heterogeneity

**Figure 4:**
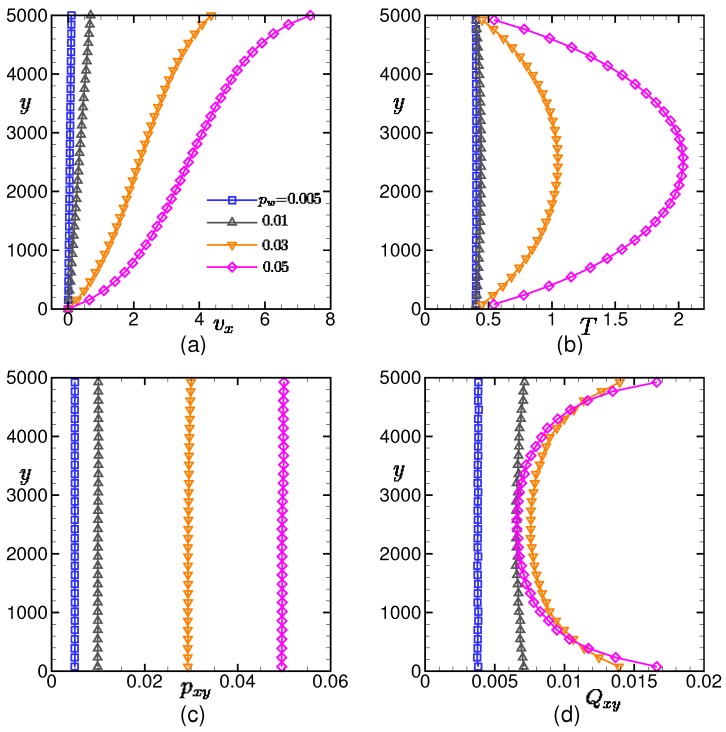
Spatial distributions of the (**a**) velocity, (**b**) temperature, (**c**) shear stress, and (**d**) bond orientation in the stationary state. The results for the different drive forces $p_{w}$ = 0.005, 0.01, 0.03, and 0.05 are shown. On the vertical axis, *y* = 5000 corresponds to the wall that is in motion, while *y* = 0 corresponds to the wall that is at rest. In each figure, the local quantities are time-averaged in the stationary state.

Figure 4 shows the spatial distribution of the velocity, temperature, shear stress, and bond orientation in the stationary state. The local quantities are time-averaged over $t=[1\times 10^{6},1.5\times 10^{6}]$ for $p_{w}<0.01$ and $t=[1.3\times 10^{6},1.5\times 10^{6}]$ for $p_{w}\geq 0.01$.

It can be seen that the local shear stresses are spatially uniform and coincide with the drive forces $p_{w}$ on the upper wall. This confirms that the momentum transport is balanced so that the macroscopic quantities are in the stationary state.

For small drive forces (i.e., $p_{w}\leq 0.01$), the temperature and bond orientation are almost uniform and the velocity is linear. On the contrary, for large drive forces (i.e., $p_{w}\geq 0.03$), the spatial variations of the temperature and bond orientation become remarkable and the velocity profile becomes S-shaped. This velocity profile indicates that the shear thinning occurs near the walls.

The temperature rises in the middle of the channel due to viscous heating, and this affects the local polymer conformation in the bond orientation. Interestingly, the local bond orientation $Q_{xy}$ monotonically increases with the drive force $p_{w}$ in the vicinities of walls, while in the middle of the channel, it behaves non-monotonically against the drive force.

**Figure 5:**
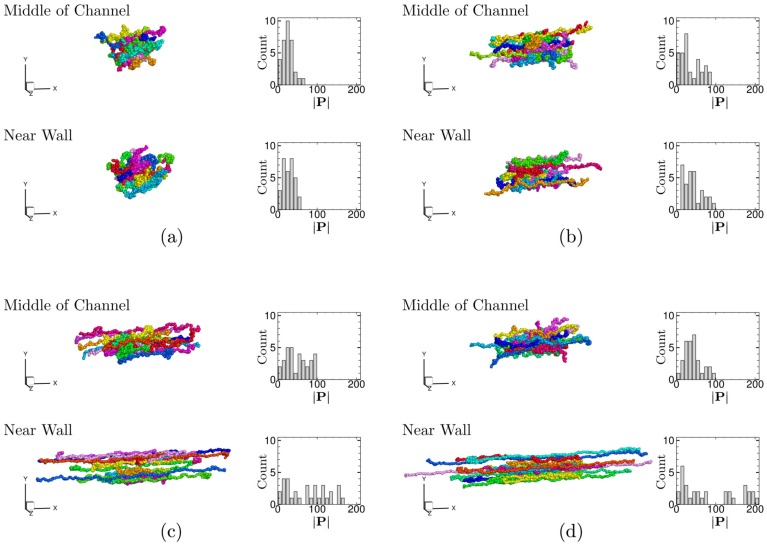
The snapshots of polymer conformation and the distributions of end-to-end distances |P| of each polymer chain in local MD cells located in the middle of the channel (*y* = 2421) and near the bottom wall (*y* = 78) at time $t=1.5\times 10^{6}$ for different drive forces, i.e., (**a**) $p_{w}$ = 0.005, (**b**) $p_{w}$ = 0.01, (**c**) $p_{w}$ = 0.03, and (**d**) $p_{w}$ = 0.09. Half of the total polymer chains contained in each MD cell, i.e., sixteen polymer chains, are plotted in each snapshot. The different colors are used to distinguish each polymer chain.

Figure 5 shows the snapshots of local polymer conformation at the middle of the channel and near the bottom wall at time $t=1.5\times 10^{6}$ for different drive forces. For small drive forces, i.e., $p_{w}=0.005$ and 0.01 (Figure 5a,b), the local polymer conformation does not significantly change between the middle of the channel and the vicinity of the wall. The polymer chains are spatially uniformly elongated in the *x*–direction as the drive force increases. It is also seen that for the small drive forces, the distribution of end-to-end distances of polymer chains |P| does not change significantly in space for each drive force, but it slightly broadens as the drive force increases.

On the other hand, when the drive force is large, i.e., $p_{w}$ = 0.03 and 0.09 (Figure 5c,d), the local polymer conformation becomes spatially heterogeneous. The polymer chains are more elongated in the vicinity of the wall than the middle of the channel. Interestingly, comparing Figure 5c,d, it is seen that the polymer chains are less elongated in the middle of the channel for the larger drive force $p_{w}$ = 0.09 than the smaller drive force $p_{w}$ = 0.03, while they are more elongated near the wall for $p_{w}$ = 0.09 than $p_{w}$ = 0.03. This can also be confirmed quantitatively from the distribution of end-to-end distances of polymer chains, i.e., the number of elongated polymers with |P|≥70 is 9 for $p_{w}$ = 0.03, while it is 5 for $p_{w}$ = 0.09. This counter-intuitive behavior will be addressed in the following subsections.

These behaviors of the local conformation of polymer chains are related to the spatial distributions of the temperature and bond orientation tensor, which are shown in Figure 4b,d, respectively. Thus, the spatial heterogeneity is generated both in the microscopic and macroscopic quantities due to the viscous heating in high-speed lubrication.

It should be noted that the increase in temperature in the middle of channel δT is enhanced when the channel width *H* becomes larger. For the Newtonian fluid with viscosity $\eta_{0}$, the relation between δT and *H* is written as $\delta T=p_{w}^{2}H^{2}/\left(8\eta_{0}\right)$. Thus, the effect of the viscous heating and the generated spatial heterogeneity become significant when the channel width is sufficiently large for a fixed drive force. The results given above clearly demonstrate that the SMD method makes it possible to address the microscopic dynamics occurring in such macroscopic-scale flow problems.

### 4.2. Gross Rheological Properties

In this subsection, we consider the gross rheological properties of the lubrication system (i.e., the apparent viscosity $\bar{\eta }$), which can be measured from the upper-wall velocity and drive force, the spatial average of the temperature $\bar{T}$, and the spatial average of the bond orientation ${\bar{Q}}_{\alpha \beta }$.

We define the gross shear rate $\dot{\Gamma }$ by $\dot{\Gamma }=v_{w}/H$, where $v_{w}$ is the upper-wall velocity. Because we consider the non-slip boundary condition, the upper-wall velocity is obtained from the velocity of polymeric liquid at y=H (i.e., $v_{w}=v_{x}\left(y=H\right)$). Then, the apparent viscosity is calculated as $\bar{\eta }=p_{w}/\dot{\Gamma }$.

**Figure 6:**
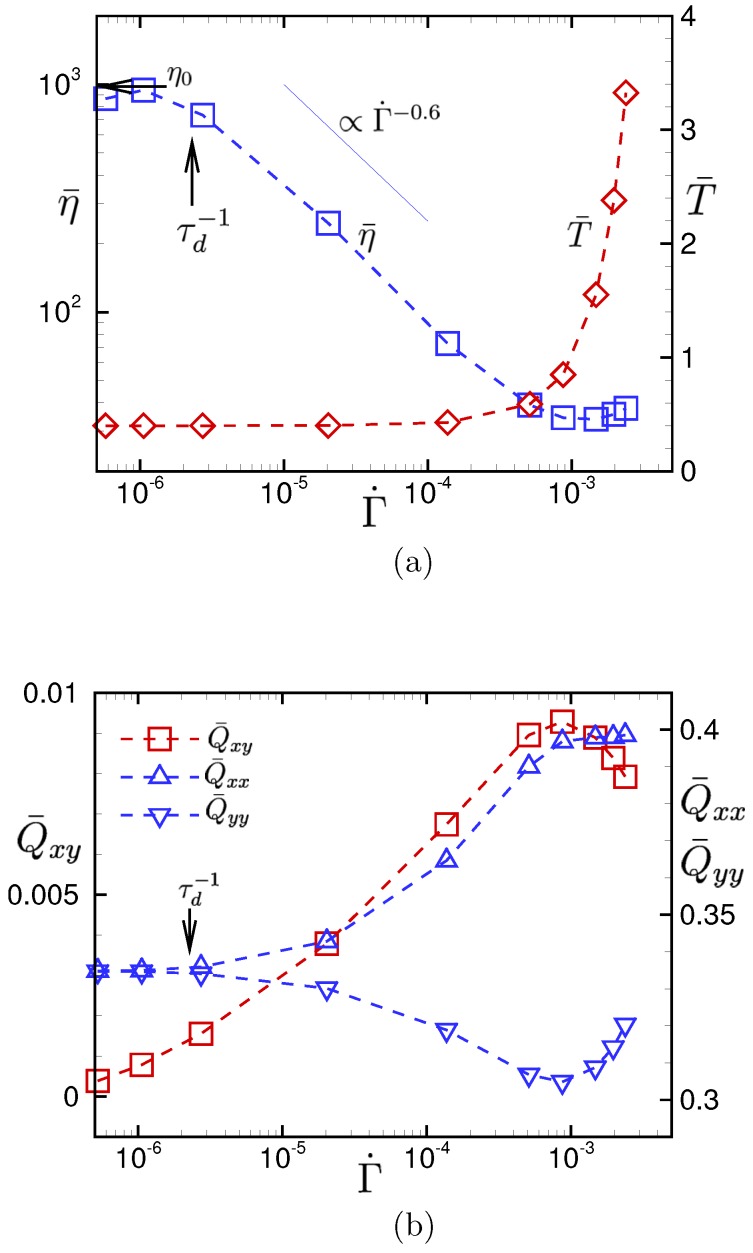
(**a**) The apparent viscosity $\bar{\eta }$ and the spatial average of the local temperature $\bar{T}$ against the gross shear rate $\dot{\Gamma }$, which is defined by the ratio of the upper-wall velocity $v_{w}$ to the channel width *H*, $\dot{\Gamma }=v_{w}/H$. (**b**) The spatial average of the local bond-orientation tensor ${\bar{Q}}_{\alpha \beta }$ against the gross shear rate $\dot{\Gamma }$. The viscosity of the model polymeric liquid in the quiescent state $\eta_{0}$ is also shown in (**a**). The rate of disengagement of entangled polymer chains in the reptation dynamics $\tau_{d}^{-1}$ is also shown in (**a**,**b**).

Figure 6 shows the apparent viscosity $\bar{\eta }$ and the spatial averages of temperature $\bar{T}$ and bond orientation of polymer chains ${\bar{Q}}_{\alpha \beta }$. The apparent viscosity approaches the intrinsic viscosity $\eta_{0}$ in the quiescent state as the gross shear rate $\dot{\Gamma }$ decreases. When the gross shear rate exceeds the inverse of the disengagement time of the entangled polymer chains (i.e., $\dot{\Gamma }>\tau_{d}^{-1}$), shear thinning behavior is observed, where the index of the power-law approximation is −0.6. However, the shear thinning behavior ends at $\dot{\Gamma }≃5\times 10^{-4}$ and, unexpectedly, even a weak shear thickening behavior is observed for $\dot{\Gamma }>10^{-3}$. The weak shear thickening behavior is obviously related to the rapid temperature rise due to the viscous heating in the large $\dot{\Gamma }$ regime.

Please note that the shear thickening behavior was not observed in the previous study for a super-cooled polymeric liquid composed of short chains, where $N_{b}$ = 10, $T_{0}$ = 0.2, and $\rho_{0}$ = 1.0 were considered [22]. For the short polymer fluid, the shear thinning behavior is further enhanced in the large-viscous heating regime. Interestingly, this qualitative difference of the thermo-rheological property between the present entangled polymer fluid and the previous short polymer fluid gives completely different velocity profiles between them.

**Figure 7:**
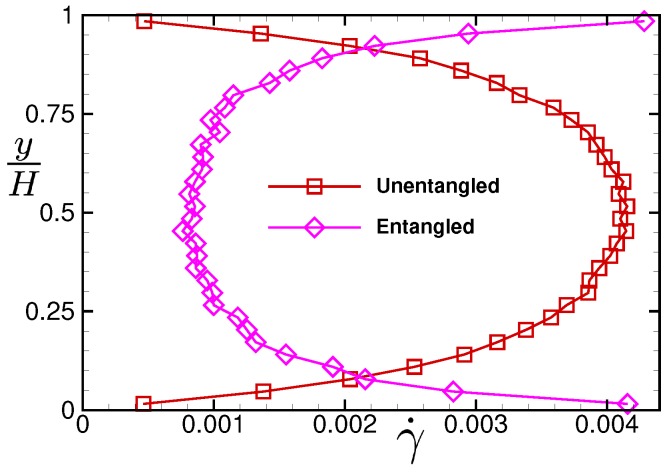
Comparison of the spatial profiles of local shear rate $\dot{\gamma }\left(y\right)$ between the present entangled polymeric liquid and the unentangled polymeric liquid studied in Ref. [22]. For the entangled one, the result for the drive force $p_{w}$ = 0.05 is plotted, while for the unentangled one, the result obtained for $p_{w}$ = 0.08, $T_{w}$ = 0.2, $\rho_{0}$ = 1.0 and *H* = 2500 is plotted. Please note that the vertical axis shows the normalized distance from the wall at rest to the wall in motion.

Figure 7 shows a comparison of the spatial profiles of shear rate $\dot{\gamma }\left(y\right)$ between the present entangled polymer fluid and the short polymer fluid studied in Ref. [22] in the strong viscous-heating regime. It is seen that, for the present polymer fluid, the local shear rate decreases in the middle of the channel because the local viscosity increases related to the increase in temperature in the strong viscous-heating regime. On the contrary, for the unentangled polymer fluid, the local shear rate increases in the middle of the channel because the local viscosity decreases related to the increase in temperature. Thus, completely different velocity profiles are generated between the present entangled polymer fluid and the short polymer fluid in the strong viscous-heating regime even in the simple one-dimensional lubrication system.

It should be noted that in previous studies [22,23,24], a severely jammed state of polymers was considered, so that the difference of the thermo-rheological property between the present entangled polymer fluid and the previous unentangled polymer fluid may be related to the initial condition of the density $\rho_{0}$ and temperature $T_{0}$. The molecular mechanism behind the different thermo-rheological property remains an open question.

The transitional behavior triggered by the viscous heating is also observed in the bond orientation of polymer chains. It can be seen from the bond-orientation tensor that polymer chains are stretched in the *x*–direction, and the xy component of the bond-orientation tensor linearly increases with the gross shear rate when the gross shear rate is in the regime $\dot{\Gamma }≲5\times 10^{-4}$. However, when $\dot{\Gamma }>10^{-3}$, the anisotropic conformation of polymer chains starts to be recovered to the uniform random conformation.

**Figure 8:**
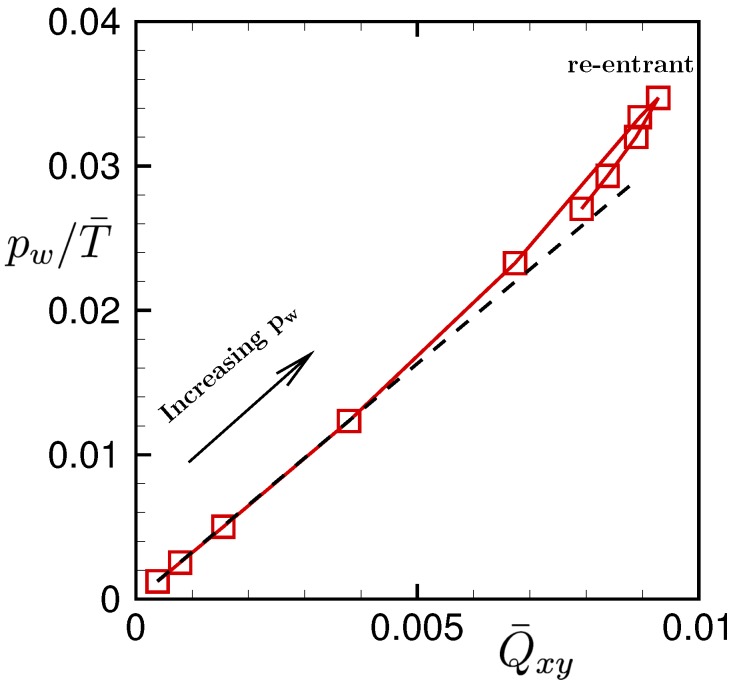
The stress–optical relation $p_{w}/\bar{T}$ vs. ${\bar{Q}}_{xy}$. The drive force $p_{w}$ increases in the direction of the arrow. The re-entrant transition of the linear stress–optical relation is observed at large gross shear rates.

Figure 8 shows the stress–optical relation for the present problem. When the drive force $p_{w}$ is small, the bond orientation ${\bar{Q}}_{xy}$ is linearly proportional to the drive force $p_{w}$, while the temperature is almost constant. Thus, a linear stress–optical relation is observed for ${\bar{Q}}_{xy}<0.005$. As the drive force $p_{w}$ increases, the results deviate from the linear relation.

However, surprisingly, the re-entrant transition of the linear stress–optical relation is observed at large drive force, where the temperature $\bar{T}$ rapidly increases and the bond orientations are recovered to be uniform and random. We note that the re-entrant transition of the linear stress–optical relation was also observed in the previous study for short polymer chains [22]. These results confirm the robustness of the linear stress–optical relation of the polymeric liquid.

The transitional behaviors stem from the competition of the effects of the flow and temperature on the polymer conformation. In the small viscous-heating regime, the temperature does not change significantly so that the conformation of polymers is only dominated by the shear flow. On the other hand, in the large viscous-heating regime, the temperature rapidly increases with the shear rate so that the thermal agitation of bead particles dominates the stretching motion of polymer chains due to the shear flow.

### 4.3. Time Evolution

**Figure 9:**
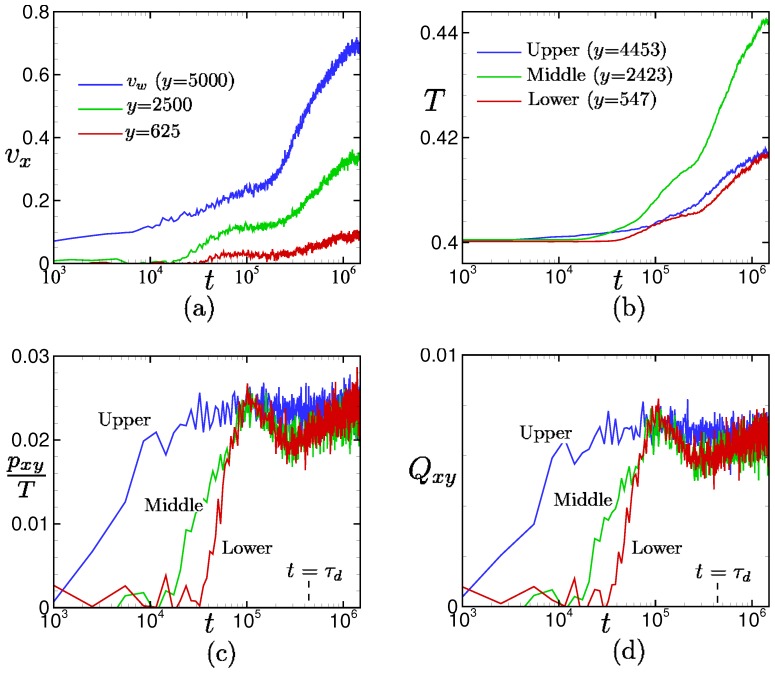
Time evolutions of (**a**) local velocity, (**b**) temperature, (**c**) shear stress, and (**d**) bond orientation for the drive force $p_{w}=0.01$. The upper-wall velocity $v_{w}$ and local velocities at *y* = 2500 and 625 are shown in (**a**). The local temperatures, local shear stresses, and local bond orientations at the upper (*y* = 4453), middle (*y* = 2423), and lower (*y* = 547) regions between the walls are shown in (**b**–**d**), respectively. The vertical dashed line on the horizontal axis shows the disengagement time of entangled polymer chains in the quiescent state.

Figure 9 shows the time evolutions of local velocities $v_{x}$, temperatures *T*, shear stresses $p_{xy}$, and bond orientations $Q_{xy}$ at different positions for the drive force $p_{w}$ = 0.01. The local shear stress and local bond orientation start to increase in the order of upper (near the moving wall) to lower (near the wall at rest) positions because the momentum is conducted from upper to lower positions, and they reach the local maxima at $t∼10^{5}$.

Yielding behaviors are clearly observed in the local shear stresses and bond orientations in $10^{5}≲t≲\tau_{d}$. After the yielding, the velocity and temperature increase rapidly, while the shear stress and bond orientation only vary slightly. It should be noted that the behaviors of the shear stress and bond orientation are quite similar to each other at each local position.

**Figure 10:**
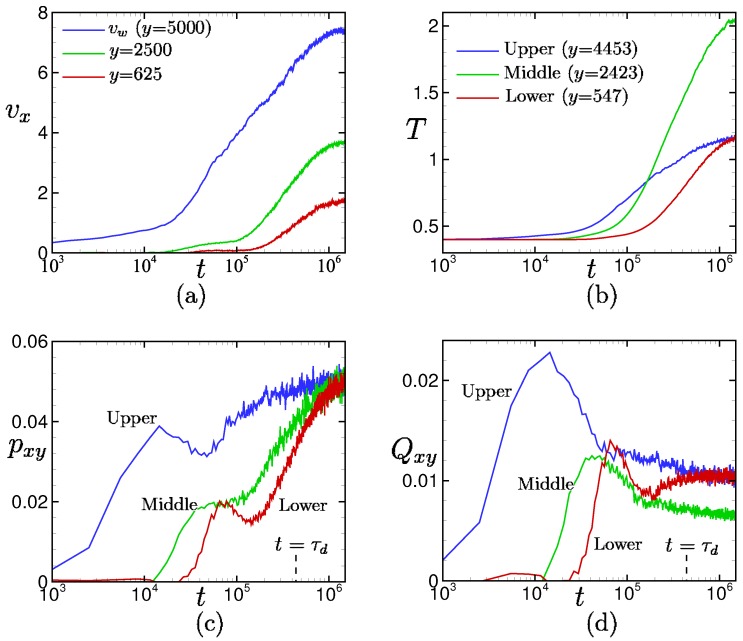
Time evolutions of (**a**) local velocity, (**b**) temperature, (**c**) shear stress, and (**d**) bond orientation for the drive force $p_{w}=0.05$. See also the caption in Figure 9.

The yielding behaviors of the shear stress and bond orientation and the rapid increase of the velocity and temperature after the yielding of the shear stress and bond orientation are also observed for the large drive force $p_{w}$ = 0.05 in Figure 10. In particular, remarkable yielding behaviors are observed in the shear stress and bond orientation at the upper and lower positions (i.e., near the walls), where the local shear rates are very large (see also Figure 4). The local maxima are reached in the order of upper to lower positions, and the times to reach the local maxima are shorter than those in the case $p_{w}$ = 0.01 (see Figure 9).

However, the yielding behaviors of the shear stress and bond orientation are quite different between $p_{w}$ = 0.01 (Figure 9) and 0.05 (Figure 10). For $p_{w}$ = 0.05, the local shear stresses increase even after the yielding, and converge to the uniform value $p_{xy}$ = $p_{w}$, while the local bond orientation decreases at the upper and middle positions but increases at the lower position.

**Figure 11:**
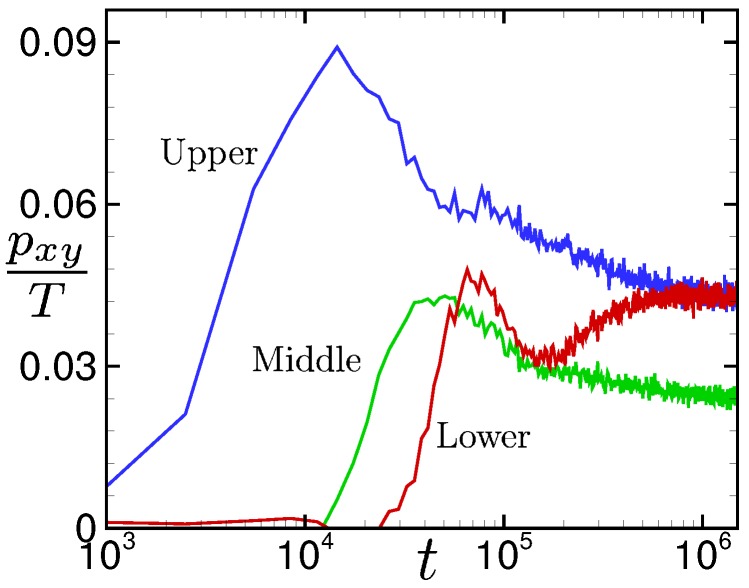
Time evolution of the local shear stress (shown in Figure 10c) divided by the local temperature (shown in Figure 10b), $p_{xy}/T$ for $p_{w}$ = 0.05.

The increase in shear stress after the yielding is highly correlated with the increase in temperature. This can be seen from Figure 11, where the time evolutions of the local shear stress divided by the local temperature $p_{xy}/T$ are shown. Surprisingly, it can be seen in comparison with Figure 10d that the time evolutions of $p_{xy}/T$ are similar to those of the bond orientations $Q_{xy}$ at each position.

**Figure 12:**
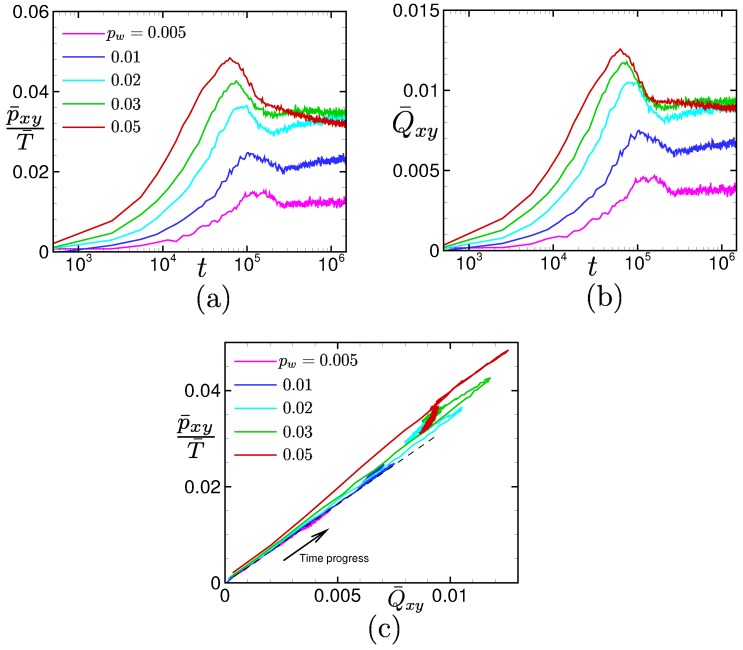
(**a**) Time evolutions of the spatial averages of the local shear stresses divided by the temperatures ${\bar{p}}_{xy}/\bar{T}$, (**b**) time evolutions of the spatial averages of the local bond orientations ${\bar{Q}}_{xy}$, and (**c**) the time evolution of the relation $\bar{p}/\bar{T}$ vs. ${\bar{Q}}_{xy}$ for different drive forces $p_{w}$ = 0.005, 0.01, 0.02, 0.03, and 0.05.

Figure 12 shows the relations between ${\bar{p}}_{xy}/\bar{T}$ and ${\bar{Q}}_{xy}$, where the spatial averages are considered, for different drive forces $p_{w}$. It can be seen that for small drive forces (i.e., $p_{w}\leq$ 0.01), both behaviors are almost synchronous. Even for large drive forces, the behaviors of ${\bar{p}}_{xy}/\bar{T}$ and ${\bar{Q}}_{xy}$ are similar to each other. In fact, it is seen from Figure 12c that the relation between ${\bar{p}}_{xy}/\bar{T}$ and ${\bar{Q}}_{xy}$ is almost linear before the yielding occurs even for large drive forces. Here, the dashed line shows the linear stress-optical relation obtained in Figure 8 (where only the stationary states are considered). Interestingly, for the large drive force $p_{w}$ = 0.05, the linear relation between ${\bar{p}}_{xy}/\bar{T}$ and ${\bar{Q}}_{xy}$ has a different coefficient from that of the linear stress-optical relation for $t≲2\times 10^{5}$. However, it gradually approaches the linear stress-optical relation in a long time period $t∼10^{6}$. These facts demonstrate that the linear stress–optical relation shown in Figure 8 approximately holds, even locally and instantaneously, at the hydrodynamic level.

## 5. Summary

We investigated the thermal lubrication of an entangled polymeric liquid in wall-driven shear flows between parallel plates by using the SMD method. The model polymeric liquid shows the characteristic behaviors of entangled polymer chains in the relaxation dynamics. That is, the transient behavior from the Rouse dynamics to the reptation dynamics is clearly observed in the stress relaxation function, as can be seen in Figure 2.

In the lubrication system, the temperature of the polymeric liquid increases in the middle of the channel due to viscous heating. The spatial profiles of both macroscopic quantities and microscopic conformation of polymers also become heterogeneous. The viscous heating becomes rapidly significant once the drive force exceeds a certain threshold value (i.e., $p_{w}≳0.02$), as can be seen in Figure 4.

The gross rheological properties are also affected by the viscous heating. As can be seen in Figure 6a that the shear viscosity shows shear thinning behavior in the weak viscous-heating regime, while in the strong viscous-heating regime, it shows a weak viscous thickening behavior related to the rapid temperature rise. This behavior is quite different from that observed in the previous studies [22,24], where a supercooled polymeric liquid composed of short chains was considered. For the unentangled polymer fluid, the shear thinning behavior was further enhanced when the temperature rapidly increased in the strong viscous-heating regime.

Although the molecular mechanism for the qualitatively different behaviors of the thermo-rheological properties between the entangled and unentangled polymer fluids in the thermal lubrication system has yet to be clarified, it is demonstrated that the qualitative difference of the thermo-rheological property gives remarkably different velocity profiles between them. For the unentangled polymer fluid, the velocity gradient becomes larger in the middle of the channel than near the wall because the shear thinning is enhanced due to the increase in temperature. On the contrary, for the entangled polymer fluid, the velocity gradient becomes smaller in the middle of the channel than near the wall because the shear thickening occurs due to the increase in temperature.

Another remarkable observation is the transitional behavior of the conformational dynamics of polymer chains shown in Figure 6b. The transition is also triggered by the viscous heating. In the weak viscous-heating regime, the conformation of polymer chains is dominated by the local shear flow, so that the anisotropy of the bond orientation grows as the drive force increases. In the strong viscous-heating regime, except in the vicinities of walls, the conformation dynamics of polymer chains is dominated by the thermal agitation of bead particles rather than the local flow field, so that the bond orientation tensor recovers the less elongated structure as the drive force increases, even though the local shear flow is further enhanced.

This counter-intuitive transitional behavior produces an interesting re-entrant transition in the stress–optical relation shown in Figure 8. The linear stress–optical relation shown in Figure 8 also approximately holds, even locally and instantaneously, at the hydrodynamic level. The robustness of the linear stress–optical relation can be observed in Figure 9, Figure 10, Figure 11 and Figure 12.

The transitional behavior of the polymer conformation against the drive force and the robustness of the stress-optical relation in the thermal lubrication system was also observed in previous studies on the unentangled polymer fluid [22,24]. In order to clarify the microscopic mechanism behind those behaviors, the investigation on the spatio-temporal dynamics of entanglement for large polymers (i.e., $N_{b}\gg$250) by using the direct measurement of entanglement, as reported in Ref. [31,32], should represent an important future research direction.
